# Supplementary material for: Cryptic diversity among Yazoo Darters (Percidae: Etheostoma raneyi) in disjunct watersheds of northern Mississippi
Source: PeerJ. 2020 May 4;8:e9014. doi: 10.7717/peerj.9014 (PMC7204820; doi:10.7717/peerj.9014)
Supplement: Supplemental Information 1 [file peerj-08-9014-s001.docx]

Table S1. Genetic data used (*cytb*) in phylogenetic analyses. Shown are locations of samples and major river drainage with GenBank accession numbers. Samples labeled “*E. raneyi* A” and “*E. raneyi* B” are from Near et al. (2011); Y.R. = Yocona River drainage, L.T.R. = Little Tallahatchie River drainage; U.T. = unnamed tributary; see Table S3 for outgroup data.

| Sample | Drainage | Stream | Latitude | Longitude | GenBank |
| --- | --- | --- | --- | --- | --- |
| SP5_Yocona | Y.R. | Splinter Creek | 34.251 | -89.642 | MT296596 |
| SP4_Yocona | Y.R. | Splinter Creek | 34.251 | -89.642 | MT296597 |
| SP10_Yocona | Y.R. | Splinter Creek | 34.251 | -89.642 | MT296598 |
| YL1_Yocona | Y.R. | Yellow Leaf Creek | 34.348 | -89.455 | MT296599 |
| 9_Yocona | Y.R. | Yellow Leaf Creek | 34.348 | -89.455 | MT296600 |
| 2_Yocona | Y.R. | Pumpkin Creek | 34.327 | -89.398 | MT296601 |
| 10_Yocona | Y.R. | Pumpkin Creek | 34.327 | -89.398 | MT296602 |
| 14_Yocona | Y.R. | Pumpkin Creek | 34.327 | -89.398 | MT296603 |
| *E. raneyi*_B | Y.R. | Pumpkin Creek | 34.327 | -89.398 | HQ128214 |
| TC2_Yocona | Y.R. | Taylor Creek | 34.293 | -89.589 | MT296604 |
| TC1_Yocona | Y.R. | Taylor Creek | 34.293 | -89.589 | MT296605 |
| X3_Yocona | Y.R. | Taylor Creek | 34.293 | -89.589 | MT296606 |
| X10_Yocona | Y.R. | Taylor Creek | 34.293 | -89.589 | MT296607 |
| X12_Yocona | Y.R. | Taylor Creek | 34.293 | -89.589 | MT296608 |
| M1_Yocona | Y.R. | Morris Creek | 34.283 | -89.544 | MT296609 |
| M3_Yocona | Y.R. | Morris Creek | 34.283 | -89.544 | MT296610 |
| M7_Yocona | Y.R. | Morris Creek | 34.283 | -89.544 | MT296611 |
| M5_Yocona | Y.R. | Morris Creek | 34.283 | -89.544 | MT296612 |
| LUT1_Otouc | Y.R. | U.T. Otoucalofa Creek | 34.125 | -89611 | MT296613 |
| U44_Otouc | Y.R. | U.T. Otoucalofa Creek | 34.125 | -89611 | MT296614 |
| U42_Otouc | Y.R. | U.T. Otoucalofa Creek | 34.125 | -89611 | MT296615 |
| U41_Otouc | Y.R. | U.T. Otoucalofa Creek | 34.125 | -89611 | MT296616 |
| GB1_Otouc | Y.R. | Gordon Branch | 34.14 | -89.549 | MT296617 |
| GB2_Otouc | Y.R. | Gordon Branch | 34.14 | -89.549 | MT296618 |
| GB4_Outoc | Y.R. | Gordon Branch | 34.14 | -89.549 | MT296619 |
| M14_Otouc | Y.R. | Mill Creek | 34.167 | -89.52 | MT296620 |
| M17_Otouc | Y.R. | Mill Creek | 34.167 | -89.52 | MT296621 |
| M20_Otouc | Y.R. | Mill Creek | 34.167 | -89.52 | MT296622 |
| M27_Otouc | Y.R. | Mill Creek | 34.167 | -89.52 | MT296623 |
| M29_Otouc | Y.R. | Mill Creek | 34.167 | -89.52 | MT296624 |
| M24_Otouc | Y.R. | Mill Creek | 34.167 | -89.52 | MT296625 |
| LJ37_Otouc | Y.R. | Johnston Creek | 34.124 | -89.641 | MT296626 |
| LJ36_Otouc | Y.R. | Johnston Creek | 34.124 | -89.641 | MT296627 |
| LJ35_Otouc | Y.R. | Johnston Creek | 34.124 | -89.641 | MT296628 |
| LJ34_Otouc | Y.R. | Johnston Creek | 34.124 | -89.641 | MT296629 |
| LJ33_Otouc | Y.R. | Johnston Creek | 34.124 | -89.641 | MT296630 |
| LJ32_Otouc | Y.R. | Johnston Creek | 34.124 | -89.641 | MT296631 |
| *E. raneyi*_A | L.T.R. | Big Spring Creek | 34.664 | -89.413 | HQ 128213 |
| T69_Talla | L.T.R. | Big Spring Creek | 34.664 | -89.413 | MT296632 |
| T68_Talla | L.T.R. | Big Spring Creek | 34.664 | -89.413 | MT296633 |
| T63-1_Talla | L.T.R. | Big Spring Creek | 34.664 | -89.413 | MT296634 |
| T61_Talla | L.T.R. | Big Spring Creek | 34.664 | -89.413 | MT296635 |
| T60_Talla | L.T.R. | Big Spring Creek | 34.664 | -89.413 | MT296636 |
| T57_Talla | L.T.R. | Big Spring Creek | 34.664 | -89.413 | MT296637 |
| T53_Talla | L.T.R. | Big Spring Creek | 34.664 | -89.413 | MT296638 |
| K5_Talla | L.T.R. | Big Spring Creek | 34.664 | -89.413 | MT296639 |
| K7_Talla | L.T.R. | Big Spring Creek | 34.664 | -89.413 | MT296640 |
| DC4_Talla | L.T.R. | Deer Creek | 34.316 | -89.785 | MT296641 |
| DC5_Talla | L.T.R. | Deer Creek | 34.316 | -89.785 | MT296642 |
| DC6_Talla | L.T.R. | Deer Creek | 34.316 | -89.785 | MT296643 |
| DC2_Talla | L.T.R. | Deer Creek | 34.316 | -89.785 | MT296644 |
| DC1_Talla | L.T.R. | Deer Creek | 34.316 | -89.785 | MT296645 |
| DC3_Talla | L.T.R. | Deer Creek | 34.316 | -89.785 | MT296646 |
| Hu13_Talla | L.T.R. | Hurricane Creek | 34.425 | -89.496 | MT296647 |
| Hu17_Talla | L.T.R. | Hurricane Creek | 34.425 | -89.496 | MT296648 |
| GM4_Talla | L.T.R. | Graham Mill Creek | 34.503 | -89.491 | MT296649 |
| GM3_Talla | L.T.R. | Graham Mill Creek | 34.503 | -89.491 | MT296650 |
| GM2_Talla | L.T.R. | Graham Mill Creek | 34.503 | -89.491 | MT296651 |
| LC80_Tippah | L.T.R. | Chewalla Creek | 34.725 | -89.305 | MT296652 |
| LC79_Tippah | L.T.R. | Chewalla Creek | 34.725 | -89.305 | MT296653 |
| UC2-5_Tippah | L.T.R. | Chewalla Creek | 34.725 | -89.305 | MT296654 |
| UC2-4_Tippah | L.T.R. | Chewalla Creek | 34.725 | -89.305 | MT296655 |
| T1_Tippah | L.T.R. | U.T. Tippah River | 34.709 | -89.256 | MT296656 |
| T2_Tippah | L.T.R. | U.T. Tippah River | 34.709 | -89.256 | MT296657 |
| CH9_Tippah | L.T.R. | Chilli Creek | 34.682 | -89.173 | MT296658 |
| CH6_Tippah | L.T.R. | Chilli Creek | 34.682 | -89.173 | MT296659 |
| CH5_Tippah | L.T.R. | Chilli Creek | 34.682 | -89.173 | MT296660 |
| CH4_Tippah | L.T.R. | Chilli Creek | 34.682 | -89.173 | MT296661 |
| CH1_Tippah | L.T.R. | Chilli Creek | 34.682 | -89.173 | MT296662 |
| YC9_Tippah | L.T.R. | Yellow Rabbit Creek | 34.819 | -89.106 | MT296663 |
| YC10_Tippah | L.T.R. | Yellow Rabbit Creek | 34.819 | -89.106 | MT296664 |
| YC13_Tippah | L.T.R. | Yellow Rabbit Creek | 34.819 | -89.106 | MT296665 |
| YY7_Tippah | L.T.R. | Yellow Rabbit Creek | 34.819 | -89.106 | MT296666 |
| YY25_Tippah | L.T.R. | Yellow Rabbit Creek | 34.819 | -89.106 | MT296667 |
| H62_Cypress | L.T.R. | Bay Springs Branch | 34.429 | -89.396 | MT296668 |
| H45_Cypress | L.T.R. | Bay Springs Branch | 34.429 | -89.396 | MT296669 |
| A25_Cypress | L.T.R. | Puskus Creek | 34.443 | -89.341 | MT296670 |
| A24_Cypress | L.T.R. | Puskus Creek | 34.443 | -89.341 | MT296671 |
| G42_Cypress | L.T.R. | Puskus Creek | 34.443 | -89.341 | MT296672 |
| G32_Cypress | L.T.R. | Puskus Creek | 34.443 | -89.341 | MT296673 |
| D39_Cypress | L.T.R. | Puskus Creek | 34.443 | -89.341 | MT296674 |
| D38_Cypress | L.T.R. | Puskus Creek | 34.443 | -89.341 | MT296675 |
| 42_Cypress | L.T.R. | Puskus Creek | 34.443 | -89.341 | MT296676 |
| 31_Cypress | L.T.R. | Puskus Creek | 34.443 | -89.341 | MT296677 |
| 30_Cypress | L.T.R. | Puskus Creek | 34.443 | -89.341 | MT296678 |
| 17_Cypress | L.T.R. | Puskus Creek | 34.443 | -89.341 | MT296679 |
| 16_Cypress | L.T.R. | Puskus Creek | 34.443 | -89.341 | MT296680 |
| 15_Cypress | L.T.R. | Puskus Creek | 34.443 | -89.341 | MT296681 |
| F40_Cypress | L.T.R. | Cypress Creek | 34.382 | -89.298 | MT296682 |
| C27_Cypress | L.T.R. | Cypress Creek | 34.382 | -89.298 | MT296683 |
| B33_Cypress | L.T.R. | Cypress Creek | 34.382 | -89.298 | MT296684 |
| 28_Cypress | L.T.R. | Cypress Creek | 34.382 | -89.298 | MT296685 |
| 27_Cypress | L.T.R. | Cypress Creek | 34.382 | -89.298 | MT296686 |
| 26_Cypress | L.T.R. | Cypress Creek | 34.382 | -89.298 | MT296687 |
| 24_Cypress | L.T.R. | Cypress Creek | 34.382 | -89.298 | MT296688 |

Table S2. Genetic data (*S7*) used in phylogenetic analyses. Shown are locations of samples and major river drainage with GenBank accession numbers. Samples labeled “*E. raneyi* A” and “*E. raneyi* B” are from Near et al. (2011); Y.R. = Yocona River drainage, L.T.R. = Little Tallahatchie River drainage; U.T. = unnamed tributary; see Table S3 for outgroup data.

| Sample | Drainage | Stream | Latitude | Longitude | GenBank |
| --- | --- | --- | --- | --- | --- |
| LJ32_Otouc | Y.R. | Johnston Creek | 34.124 | -89.641 | MT296689 |
| LJ33_Otouc | Y.R. | Johnston Creek | 34.124 | -89.641 | MT296690 |
| LJ34_Otouc | Y.R. | Johnston Creek | 34.124 | -89.641 | MT296691 |
| LJ35_Otouc | Y.R. | Johnston Creek | 34.124 | -89.641 | MT296692 |
| LJ36_Otouc | Y.R. | Johnston Creek | 34.124 | -89.641 | MT296693 |
| LJ37_Otouc | Y.R. | Johnston Creek | 34.124 | -89.641 | MT296694 |
| M18_Otouc | Y.R. | Mill Creek | 34.167 | -89.52 | MT296695 |
| M19_Otouc | Y.R. | Mill Creek | 34.167 | -89.52 | MT296696 |
| M20_Otouc | Y.R. | Mill Creek | 34.167 | -89.52 | MT296697 |
| M21_Otouc | Y.R. | Mill Creek | 34.167 | -89.52 | MT296698 |
| M24_Otouc | Y.R. | Mill Creek | 34.167 | -89.52 | MT296699 |
| M29_Otouc | Y.R. | Mill Creek | 34.167 | -89.52 | MT296700 |
| U41_Otouc | Y.R. | U.T. Otoucalofa Creek | 34.125 | -89611 | MT296701 |
| U42_Otouc | Y.R. | U.T. Otoucalofa Creek | 34.125 | -89611 | MT296702 |
| U44_Otouc | Y.R. | U.T. Otoucalofa Creek | 34.125 | -89611 | MT296703 |
| GB1_Otouc | Y.R. | Gordon Branch | 34.14 | -89.549 | MT296704 |
| GB2_Otouc | Y.R. | Gordon Branch | 34.14 | -89.549 | MT296705 |
| M3_Yocona | Y.R. | Morris Creek | 34.283 | -89.544 | MT296706 |
| M4_Yocona | Y.R. | Morris Creek | 34.283 | -89.544 | MT296707 |
| M5_Yocona | Y.R. | Morris Creek | 34.283 | -89.544 | MT296708 |
| M6_Yocona | Y.R. | Morris Creek | 34.283 | -89.544 | MT296709 |
| YL1_Yocona | Y.R. | Yellow Leaf Creek | 34.348 | -89.455 | MT296710 |
| SL2_Yocona | Y.R. | Splinter Creek | 34.251 | -89.642 | MT296711 |
| SL4_Yocona | Y.R. | Splinter Creek | 34.251 | -89.642 | MT296712 |
| SL5_Yocona | Y.R. | Splinter Creek | 34.251 | -89.642 | MT296713 |
| Sp10_Yocona | Y.R. | Splinter Creek | 34.251 | -89.642 | MT296714 |
| TC1_Yocona | Y.R. | Taylor Creek | 34.293 | -89.589 | MT296715 |
| TC2_Yocona | Y.R. | Taylor Creek | 34.293 | -89.589 | MT296716 |
| X12_Yocona | Y.R. | Taylor Creek | 34.293 | -89.589 | MT296717 |
| X3_Yocona | Y.R. | Taylor Creek | 34.293 | -89.589 | MT296718 |
| X10_Yocona | Y.R. | Taylor Creek | 34.293 | -89.589 | MT296719 |
| *E. raneyi* B | Y.R. | Pumpkin Creek | 34.327 | -89.398 | HQ128451 |
| 16_Cypress | L.T.R. | Puskus Creek | 34.443 | -89.341 | MT296720 |
| 17_Cypress | L.T.R. | Puskus Creek | 34.443 | -89.341 | MT296721 |
| 31_Cypress | L.T.R. | Puskus Creek | 34.443 | -89.341 | MT296722 |
| 42_Cypress | L.T.R. | Puskus Creek | 34.443 | -89.341 | MT296723 |
| 22_Cypress | L.T.R. | Puskus Creek | 34.443 | -89.341 | MT296724 |
| 15_Cypress | L.T.R. | Puskus Creek | 34.443 | -89.341 | MT296725 |
| C27_Cypress | L.T.R. | Cypress Creek | 34.382 | -89.298 | MT296726 |
| H62_Cypress | L.T.R. | Bay Springs Branch | 34.429 | -89.396 | MT296727 |
| 26_Cypress | L.T.R. | Cypress Creek | 34.382 | -89.298 | MT296728 |
| 24_Cypress | L.T.R. | Cypress Creek | 34.382 | -89.298 | MT296729 |
| 27_Cypress | L.T.R. | Cypress Creek | 34.382 | -89.298 | MT296730 |
| F49_Cypress | L.T.R. | Cypress Creek | 34.382 | -89.298 | MT296731 |
| B33_Cypress | L.T.R. | Cypress Creek | 34.382 | -89.298 | MT296732 |
| *E. raneyi* A | L.T.R. | Big Spring Creek | 34.664 | -89.413 | HQ128450 |
| T52_Talla | L.T.R. | Big Spring Creek | 34.664 | -89.413 | MT296733 |
| T53_Talla | L.T.R. | Big Spring Creek | 34.664 | -89.413 | MT296734 |
| T54_Talla | L.T.R. | Big Spring Creek | 34.664 | -89.413 | MT296735 |
| T55_Talla | L.T.R. | Big Spring Creek | 34.664 | -89.413 | MT296736 |
| T57_Talla | L.T.R. | Big Spring Creek | 34.664 | -89.413 | MT296737 |
| T58_Talla | L.T.R. | Big Spring Creek | 34.664 | -89.413 | MT296738 |
| T59_Talla | L.T.R. | Big Spring Creek | 34.664 | -89.413 | MT296739 |
| T61_Talla | L.T.R. | Big Spring Creek | 34.664 | -89.413 | MT296740 |
| T68_Talla | L.T.R. | Big Spring Creek | 34.664 | -89.413 | MT296741 |
| T69_Talla | L.T.R. | Big Spring Creek | 34.664 | -89.413 | MT296742 |
| K5_Talla | L.T.R. | Big Spring Creek | 34.664 | -89.413 | MT296743 |
| K2_Talla | L.T.R. | Big Spring Creek | 34.664 | -89.413 | MT296744 |
| DC3_Talla | L.T.R. | Deer Creek | 34.316 | -89.785 | MT296745 |
| DC1_Talla | L.T.R. | Deer Creek | 34.316 | -89.785 | MT296746 |
| DC2_Talla | L.T.R. | Deer Creek | 34.316 | -89.785 | MT296747 |
| DC6_Talla | L.T.R. | Deer Creek | 34.316 | -89.785 | MT296748 |
| DC5_Talla | L.T.R. | Deer Creek | 34.316 | -89.785 | MT296749 |
| DC4_Talla | L.T.R. | Deer Creek | 34.316 | -89.785 | MT296750 |
| GM2_Talla | L.T.R. | Graham Mill Creek | 34.503 | -89.491 | MT296751 |
| GM3_Talla | L.T.R. | Graham Mill Creek | 34.503 | -89.491 | MT296752 |
| GM4_Talla | L.T.R. | Graham Mill Creek | 34.503 | -89.491 | MT296753 |
| Hu13_Talla | L.T.R. | Hurricane Creek | 34.425 | -89.496 | MT296754 |
| Hu15_Talla | L.T.R. | Hurricane Creek | 34.425 | -89.496 | MT296755 |
| HU17_Talla | L.T.R. | Hurricane Creek | 34.425 | -89.496 | MT296756 |
| LC80_Tippah | L.T.R. | Chewalla Creek | 34.725 | -89.305 | MT296757 |
| UC2-4_Tippah | L.T.R. | Chewalla Creek | 34.725 | -89.305 | MT296758 |
| UC2-5_Tippah | L.T.R. | Chewalla Creek | 34.725 | -89.305 | MT296759 |
| CH2_Tippah | L.T.R. | Chilli Creek | 34.682 | -89.173 | MT296760 |
| CH6_Tippah | L.T.R. | Chilli Creek | 34.682 | -89.173 | MT296761 |
| CH9_Tippah | L.T.R. | Chilli Creek | 34.682 | -89.173 | MT296762 |
| CH1_Tippah | L.T.R. | Chilli Creek | 34.682 | -89.173 | MT296763 |
| T6_Tippah | L.T.R. | U.T. Tippah River | 34.709 | -89.256 | MT296764 |
| T9a_Tippah | L.T.R. | U.T. Tippah River | 34.709 | -89.256 | MT296765 |
| YY7_Tippah | L.T.R. | Yellow Rabbit Creek | 34.819 | -89.106 | MT296766 |
| YY25_Tippah | L.T.R. | Yellow Rabbit Creek | 34.819 | -89.106 | MT296767 |
| YY24_Tippah | L.T.R. | Yellow Rabbit Creek | 34.819 | -89.106 | MT296768 |
| YY20_Tippah | L.T.R. | Yellow Rabbit Creek | 34.819 | -89.106 | MT296769 |

Table S3. GenBank accession numbers for outgroup sequences used in phylogenetic analyses.

| Sample | GenBank |
| --- | --- |
| *Etheostoma zonistium* A | HQ128255 |
| *E. zonistium* B | HQ128256 |
| *E. cyanoprosopum* | HQ128100 |
| *E. cervus* | HQ128102 |
| *E. pyrrhogaster* | HQ128207 |
| *E. nigrum* | AF 183945 |
| *Percina sciera* | HQ 128270 |

Table S4. Genetic sequence data (*cytb*) used for estimating uncorrected pairwise genetic distances among snubnose darters. GenBank accession numbers are given for all sources except data stored on Dryad (https://datadryad.org/) from Kozal et al. (2017); these sequences are labeled as in Kozal et al. (2017). This data set was added to the Yazoo Darter sequences from Table S1 for estimating genetic distances.

| **Species** | **Citation** | **GenBank/Dryad** |
| --- | --- | --- |
| *Etheostoma bellator* | Near et al., 2011 | HQ128088 |
| *E. bellator* | Porterfield 1998 | AF288425 |
| *E*. cf. *bellator* | Near et al., 2011 | HQ128087 |
| *E.* cf. *bellator* | Near et al., 2011 | HQ128086 |
| *E.* cf. *bellator* | Near et al., 2011 | JF742808 |
| *E.* cf. *bellator* | Near et al., 2011 | JF742807 |
| *E. brevirostrum* | Porterfield 1998 | AF288428 |
| *E.* cf. *brevirostrum* | Near et al., 2011 | HQ128097 |
| *E*. cf. *brevirostrum* | Near et al., 2011 | HQ128096 |
| *E. cervus* | Near et al., 2011 | HQ128101.1 |
| *E. cervus* | Near et al., 2011 | HQ128102 |
| *E. cervus* | Powers & Warren 2009 | FJ423444 |
| *E. cervus* | Powers & Warren 2009 | FJ423443 |
| *E. cervus* | Powers & Warren 2009 | FJ423442 |
| *E. cervus* | Powers & Warren 2009 | FJ423441 |
| *E. cervus* | Kozal et al., 2017 | A |
| *E. cervus* | Kozal et al., 2017 | B |
| *E. cervus* | Kozal et al., 2017 | E |
| *E. chermocki* | Near et al., 2011 | HQ128103 |
| *E. chermocki* | Porterfield 1998 | AF288429 |
| *E. colorosum* | Near et al., 2011 | HQ128113 |
| *E. colorosum* | Near et al., 2011 | HQ128112 |
| *E. colorosum* | Porterfield 1998 | AF288430 |
| *E. coosae* | Near et al., 2011 | HQ128115 |
| *E. coosae* | Near et al., 2011 | HQ128114 |
| *E. coosae* | Porterfield 1998 | AF288431 |
| *E. cyanoprosopum* | Near et al., 2011 | HQ128100 |
| *E. cyanoprosopum* | Kozal et al., 2017 | CL |
| *E. cyanoprosopum* | Kozal et al., 2017 | AF |
| *E. cyanoprosopum* | Kozal et al., 2017 | AD |
| *E. cyanoprosopum* | Kozal et al., 2017 | X |
| *E. cyanoprosopum* | Kozal et al., 2017 | Z |
| *E. cyanoprosopum* | Kozal et al., 2017 | AB |
| *E. duryi* | Near et al., 2011 | HQ128124 |
| *E. duryi* | Near et al., 2011 | HQ128123 |
| *E. duryi* | Porterfield 1998 | AF288432 |
| *E. etnieri* | Near et al., 2011 | HQ128128 |
| *E. etnieri* | Porterfield 1998 | AF288433 |
| *E. flavum* | Near et al., 2011 | HQ128133 |
| *E. flavum* | Near et al., 2011 | HQ128132 |
| *E. flavum* | Porterfield 1998 | AF288434 |
| *E. flavum* | Switzer 2004 | AY964714 |
| *E. lachneri* | Near et al., 2011 | HQ128155.1 |
| *E. lachneri* | Near et al., 2011 | HQ128154.1 |
| *E. lachneri* | Porterfield 1998 | AF288436 |
| *E. lachneri* | Powers & Warren 2009 | FJ423445 |
| *E. pyrrhogaster* | Near et al., 2011 | HQ128206.1 |
| *E. pyrrhogaster* | Near et al., 2011 | HQ128207 |
| *E. pyrrhogaster* | Porterfield 1998 | AF288438 |
| *E. pyrrhogaster* | Powers & Warren 2009 | FJ423440 |
| *E. pyrrhogaster* | Powers & Warren 2009 | FJ423439 |
| *E. pyrrhogaster* | Powers & Warren 2009 | FJ423438 |
| *E. pyrrhogaster* | Powers & Warren 2009 | FJ423437 |
| *E. pyrrhogaster* | Kozal et al., 2017 | A |
| *E. pyrrhogaster* | Kozal et al., 2017 | B |
| *E. pyrrhogaster* | Kozal et al., 2017 | E |
| *E. ramseyi* | Near et al., 2011 | HQ128212 |
| *E. ramseyi* | Near et al., 2011 | HQ128211 |
| *E. ramseyi* | Porterfield 1998 | AF288440 |
| *E. scotti* | Near et al., 2011 | HQ128218 |
| *E. scotti* | Porterfield 1998 | AF288443 |
| *E. tallapoosae* | Near et al., 2011 | HQ128242 |
| *E. tallapoosae* | Near et al., 2011 | HQ128241 |
| *E. zonistium* | Near et al., 2011 | HQ128255 |
| *E. zonistium* | Near et al., 2011 | HQ128256 |
| *E. zonistium* | Kozal et al., 2017 | AD |
| *E. zonistium* | Kozal et al., 2017 | AE |
| *E. zonistium* | Kozal et al., 2017 | AF |
| *E. zonistium* | Kozal et al., 2017 | AA |
| *E. zonistium* | Kozal et al., 2017 | AB |
| *E. zonistium* | Kozal et al., 2017 | S |
| *E. zonistium* | Kozal et al., 2017 | T |
| *E. zonistium* | Kozal et al., 2017 | BU |
| *E. zonistium* | Kozal et al., 2017 | BW |
| *E. zonistium* | Kozal et al., 2017 | CL |
| *E. zonistium* | Kozal et al., 2017 | AQ |
| *E. zonistium* | Kozal et al., 2017 | AT |
| *E. zonistium* | Kozal et al., 2017 | AW |
| *E. zonistium* | Kozal et al., 2017 | AX |
| *E. zonistium* | Kozal et al., 2017 | AY |
| *E. zonistium* | Kozal et al., 2017 | AZ |
| *E. zonistium* | Kozal et al., 2017 | BC |
| *E. zonistium* | Kozal et al., 2017 | BD |
| *E. zonistium* | Kozal et al., 2017 | BE |
| *E. zonistium* | Kozal et al., 2017 | BG |
| *E. zonistium* | Kozal et al., 2017 | BH |
| *E. zonistium* | Kozal et al., 2017 | BI |
| *E. zonistium* | Kozal et al., 2017 | B |
| *E. zonistium* | Kozal et al., 2017 | G |
| *E. zonistium* | Kozal et al., 2017 | K |
| *E. zonistium* | Kozal et al., 2017 | L |
| *E. zonistium* | Kozal et al., 2017 | DJ |
| *E. zonistium* | Kozal et al., 2017 | DK |
| *E. zonistium* | Kozal et al., 2017 | DL |
| *E. zonistium* | Kozal et al., 2017 | O |
| *E. zonistium* | Kozal et al., 2017 | P |
| *E. zonistium* | Kozal et al., 2017 | Q |
| *E. zonistium* | Kozal et al., 2017 | R |
| *E.* cf. *zonistium* | Porterfield 1998 | AF288450 |
| *E.* cf. *zonistium* | Kozal et al., 2017 | DU |
| *E.* cf. *zonistium* | Kozal et al., 2017 | DX |
| *E.* cf. *zonistium* | Kozal et al., 2017 | EB |
| *E.* cf. *zonistium* | Kozal et al., 2017 | ED |
| *E*. cf. *zonistium* | Kozal et al., 2017 | EE |
| *E*. cf. *zonistium* | Kozal et al., 2017 | EG |
| *E*. cf. *zonistium* | Kozal et al., 2017 | EH |
| *E*. cf. *zonistium* | Kozal et al., 2017 | EI |
| *E*. cf. *zonistium* | Kozal et al., 2017 | EJ |
| *E.* cf. *zonistium* | Kozal et al., 2017 | EK |
| *E. simoterum* | Near et al., 2011 | HQ128226 |
| *E. zonale* | Near et al., 2011 | HQ128252 |
| *E. nigrum* | Near, Porterfield & Page 2000 | AF183945 |
| *Percina sciera* | Near et al., 2011 | HQ 128270 |

Table S5. Uncorrected pairwise genetic distances (*cytb*) among snubnose darters (MEGA analyses) are shown. The number of base differences per site from averaging over all sequence pairs between groups are shown. All positions containing gaps and missing data were eliminated yielding a total of 1047 of 1140 bp used for estimates. Labels for undescribed species follows Jelks et al. (2008), Y.R. = Yocona River drainage, L.T.R. = Little Tallahatchie River drainage.

|  | Y.R. | L.T.R. | *E. cyanoprosopum* | | *E. cervus* |
| --- | --- | --- | --- | --- | --- |
| Yazoo Darter, Y.R. |  |  |  | |  |
| Yazoo Darter, L.T.R. | 0.75 |  |  | |  |
| *E. cyanoprosopum* | 8.84 | 9.24 |  | |  |
| *E. cervus* | 8.45 | 8.97 | 4.86 | |  |
| *E. pyrrhogaster* | 8.61 | 9.04 | 4.84 | | 0.86 |
| *E. nigrum* | 14.90 | 15.05 | 15.17 | | 15.20 |
| *Percina sciera* | 16.91 | 16.81 | 17.11 | | 17.46 |
| *E. lachneri* | 8.60 | 9.18 | 9.16 | | 9.11 |
| *E.* cf. *bellator*, Sipsey | 10.29 | 10.40 | 11.02 | | 10.99 |
| *E.* cf. *brevirostrum*, Conasauga | 8.79 | 8.71 | 9.51 | | 9.41 |
| *E.* cf. *brevirostrum*, Amicalola | 7.84 | 7.95 | 8.20 | | 8.17 |
| *E. ramseyi* | 8.53 | 8.83 | 9.54 | | 9.17 |
| *E. bellator* | 8.50 | 8.99 | 9.84 | | 9.48 |
| *E.* cf. *bellator*, Locust Fork | 9.70 | 10.18 | 10.24 | | 9.80 |
| *E. colorosum* | 7.20 | 7.37 | 8.97 | | 9.25 |
| *E. etnieri* | 9.94 | 10.47 | 10.06 | | 9.69 |
| *E. chermocki* | 8.22 | 8.71 | 9.62 | | 9.29 |
| *E. tallapoosae* | 8.22 | 8.26 | 8.77 | | 8.21 |
| *E. flavum* | 13.52 | 13.62 | 13.33 | | 14.55 |
| *E. duryi* | 13.38 | 13.57 | 11.97 | | 13.90 |
| *E. coosae* | 12.52 | 12.96 | 13.44 | | 14.54 |
| *E. scotti* | 12.80 | 13.00 | 12.55 | | 14.01 |
| *E. brevirostrum*, Shoal Creek | 8.79 | 8.71 | 9.78 | | 9.60 |
| *E.* cf. *zonistium*, Hatchie River | 8.33 | 8.64 | 4.75 | | 0.50 |
| *E. simoterum* | 14.33 | 14.24 | 15.01 | | 15.04 |
| *E. zonistium* | 7.61 | 8.07 | 4.25 | | 1.42 |
| *E. zonale* | 15.00 | 15.09 | 15.15 | | 15.00 |
|  |  |  |  | |  |
|  | *E. pyrrhogaster* | *E. nigrum* | *Percina sciera* | | *E. lachneri* |
| *E. pyrrhogaster* |  |  |  | |  |
| *E. nigrum* | 15.19 |  |  | |  |
| *Percina sciera* | 17.47 | 16.33 |  | |  |
| *E. lachneri* | 9.10 | 15.26 | 15.71 | |  |
| *E.* cf. *bellator*, Sipsey | 10.84 | 15.98 | 17.03 | | 6.15 |
| *E.* cf. *brevirostrum*, Conasauga | 9.46 | 15.09 | 17.77 | | 8.05 |
| *E.* cf. *brevirostrum*, Amicalola | 8.41 | 13.94 | 17.00 | | 6.33 |
| *E. ramseyi* | 9.37 | 14.84 | 17.00 | | 7.89 |
| *E. bellator* | 9.39 | 15.38 | 15.85 | | 5.73 |
| *E.* cf. *bellator*, Locust Fork | 9.81 | 15.43 | 15.62 | | 5.56 |
| *E. colorosum* | 8.99 | 14.61 | 16.49 | | 5.35 |
| *E. etnieri* | 9.82 | 15.09 | 15.76 | | 8.95 |
| *E. chermocki* | 9.20 | 15.00 | 15.57 | | 5.16 |
| *E. tallapoosae* | 8.22 | 14.14 | 16.71 | | 7.88 |
| *E. flavum* | 14.48 | 15.74 | 16.64 | | 13.96 |
| *E. duryi* | 14.20 | 16.27 | 17.00 | | 13.28 |
| *E. coosae* | 14.53 | 15.57 | 16.91 | | 13.58 |
| *E. scotti* | 14.01 | 16.14 | 16.76 | | 12.56 |
| *E. brevirostrum*, Shoal Creek | 9.68 | 15.47 | 17.86 | | 8.52 |
| *E.* cf. *zonistium*, Hatchie River | 0.72 | 14.86 | 17.30 | | 9.04 |
| *E. simoterum* | 15.42 | 16.81 | 17.10 | | 14.18 |
| *E. zonistium* | 1.44 | 14.71 | 16.81 | | 8.61 |
| *E. zonale* | 14.56 | 16.71 | 17.48 | | 15.02 |
|  |  |  |  | |  |
|  | Sipsey | Conasauga | Amicalola | | *E. ramseyi* |
| *E.* cf. *bellator*, Sipsey |  |  |  | |  |
| *E.* cf. *brevirostrum*, Conasauga | 9.84 |  |  | |  |
| *E.* cf. *brevirostrum*, Amicalola | 8.21 | 3.15 |  | |  |
| *E. ramseyi* | 9.39 | 7.20 | 6.62 | |  |
| *E. bellator* | 6.24 | 8.79 | 7.45 | | 8.63 |
| *E.* cf. *brevirostrum*, Locust Fork | 6.57 | 9.22 | 7.69 | | 8.96 |
| *E. colorosum* | 6.75 | 7.70 | 6.21 | | 7.64 |
| *E. etnieri* | 10.22 | 10.51 | 8.98 | | 10.16 |
| *E. chermocki* | 6.05 | 8.60 | 7.07 | | 8.25 |
| *E. tallapoosae* | 9.42 | 7.26 | 5.54 | | 7.26 |
| *E. flavum* | 14.96 | 14.04 | 13.49 | | 13.55 |
| *E. duryi* | 14.04 | 12.83 | 12.13 | | 13.79 |
| *E. coosae* | 13.53 | 13.85 | 13.21 | | 14.04 |
| *E. scotti* | 13.36 | 13.47 | 12.89 | | 12.97 |
| *E. brevirostrum*, Shoal Creek | 10.12 | 1.05 | 3.63 | | 7.67 |
| *E.* cf. *zonistium*, Hatchie River | 11.02 | 9.32 | 8.08 | | 9.06 |
| *E. simoterum* | 14.52 | 14.90 | 13.94 | | 14.55 |
| *E. zonistium* | 10.69 | 9.03 | 7.78 | | 8.95 |
| *E. zonale* | 15.28 | 14.23 | 15.00 | | 14.45 |
|  |  |  |  | |  |
|  | *E. bellator* | Locust Fork | *E. colorosum* | | *E. etnieri* |
| *E. bellator* |  |  |  | |  |
| *E.* cf. *bellator*, Locust Fork | 5.11 |  |  | |  |
| *E. colorosum* | 5.83 | 6.03 |  | |  |
| *E. etnieri* | 9.26 | 8.83 | 7.99 | |  |
| *E. chermocki* | 0.57 | 4.92 | 5.64 | | 9.07 |
| *E. tallapoosae* | 8.31 | 8.93 | 7.13 | | 9.84 |
| *E. flavum* | 13.92 | 13.42 | 13.37 | | 14.23 |
| *E. duryi* | 13.79 | 13.51 | 13.91 | | 13.79 |
| *E. coosae* | 14.07 | 12.94 | 13.27 | | 13.50 |
| *E. scotti* | 13.32 | 12.61 | 12.46 | | 14.09 |
| *E. brevirostrum,* Shoal Creek | 8.88 | 9.89 | 7.86 | | 11.08 |
| *E.* cf. *zonistium*, Hatchie River | 9.38 | 9.81 | 9.15 | | 9.62 |
| *E. simoterum* | 14.33 | 13.90 | 14.29 | | 14.52 |
| *E. zonistium* | 8.93 | 9.64 | 8.45 | | 9.07 |
| *E. zonale* | 15.28 | 15.81 | 14.84 | | 15.28 |
|  |  |  |  | |  |
|  | *E. chermocki* | *E. tallapoosae* | | *E. flavum* | *E. duryi* |
| *E. chermocki* |  |  |  | |  |
| *E. tallapoosae* | 7.93 |  |  | |  |
| *E. flavum* | 13.59 | 13.54 |  | |  |
| *E. duryi* | 13.40 | 13.09 | 8.13 | |  |
| *E. coosae* | 13.72 | 12.73 | 12.88 | | 13.31 |
| *E. scotti* | 12.89 | 12.27 | 13.49 | | 12.56 |
| *E. brevirostrum*, Shoal Creek | 8.69 | 7.26 | 14.30 | | 13.18 |
| *E.* cf. *zonistium*, Hatchie River | 9.19 | 7.91 | 14.37 | | 13.96 |
| *E. simoterum* | 14.14 | 13.47 | 14.83 | | 13.94 |
| *E. zonistium* | 8.74 | 7.61 | 14.10 | | 13.80 |
| *E. zonale* | 14.80 | 14.14 | 15.14 | | 15.66 |
|  |  |  |  | |  |
|  | *E. coosae* | *E. scotti* | *E. brevirostrum* | | Hatchie River |
| *E. scotti* | 9.54 |  |  | |  |
| *E. brevirostrum*, Shoal Creek | 14.10 | 13.37 |  | |  |
| *E.* cf. *zonistium*, Hatchie River | 14.49 | 13.91 | 9.51 | |  |
| *E. simoterum* | 15.44 | 15.19 | 14.71 | | 15.01 |
| *E. zonistium* | 14.01 | 13.43 | 9.22 | | 1.29 |
| *E. zonale* | 16.17 | 16.38 | 14.52 | | 14.73 |
|  |  |  |  | |  |
|  | *E. simoterum* | *E. zonistium* |  | |  |
| *E. simoterum* |  |  |  | |  |
| *E. zonistium* | 15.03 |  |  | |  |
| *E. zonale* | 12.80 | 14.76 |  | |  |


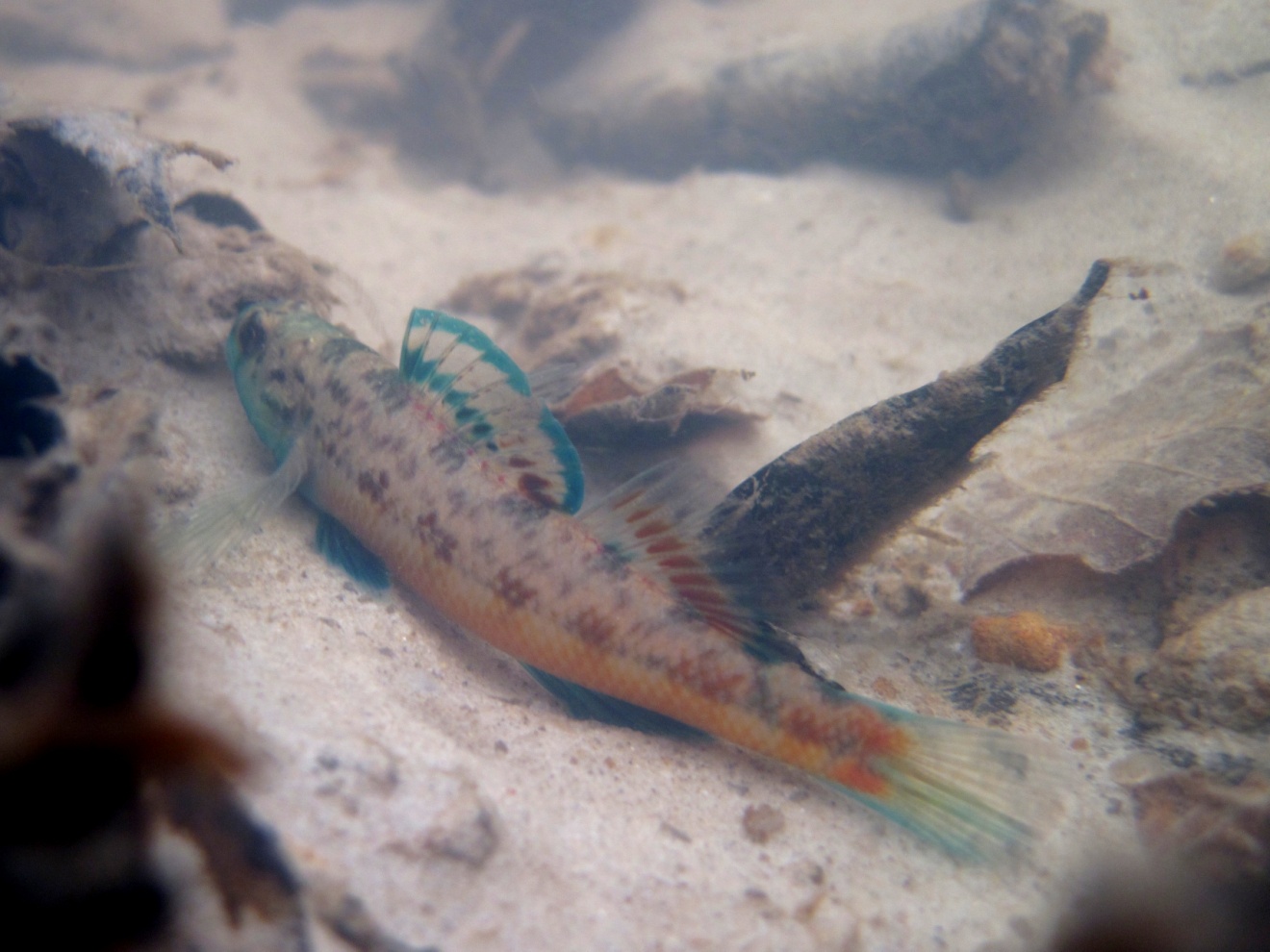


Figure S1. Male Yazoo Darter in Puskus Creek, Little Tallahatchie River drainage. Photo credit: Ken Sterling.


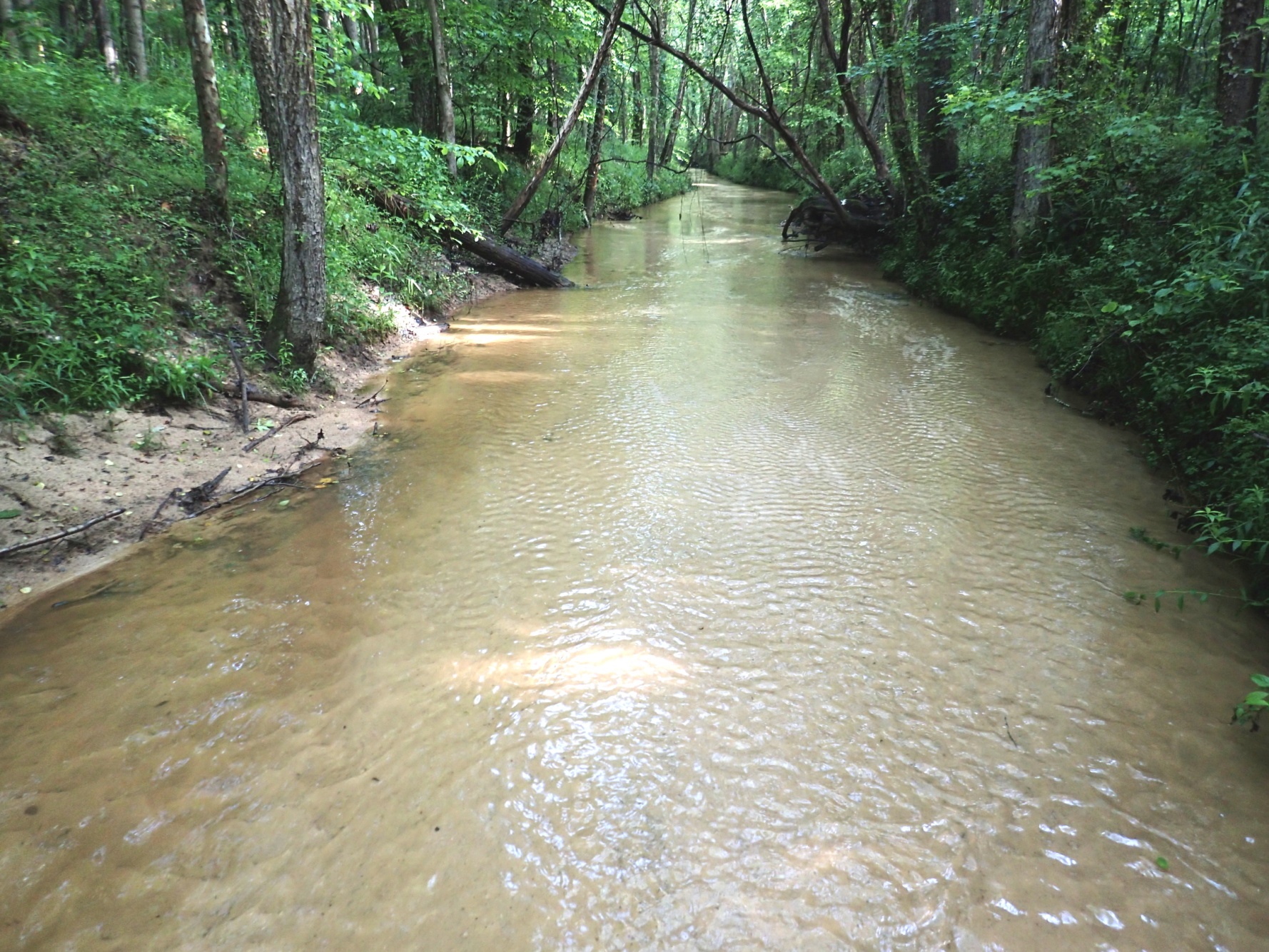


Figure S2. Photograph of Puskus Creek (Little Tallahatchie River drainage) showing typical stream habitat within the distribution of the Yazoo Darter; the stream reach pictured once ran through agricultural fields and is now recovering from channelization and incisement but still lacks stable structure (i.e., wood) used by aquatic organisms including the Yazoo Darter. Photo credit: Ken Sterling.


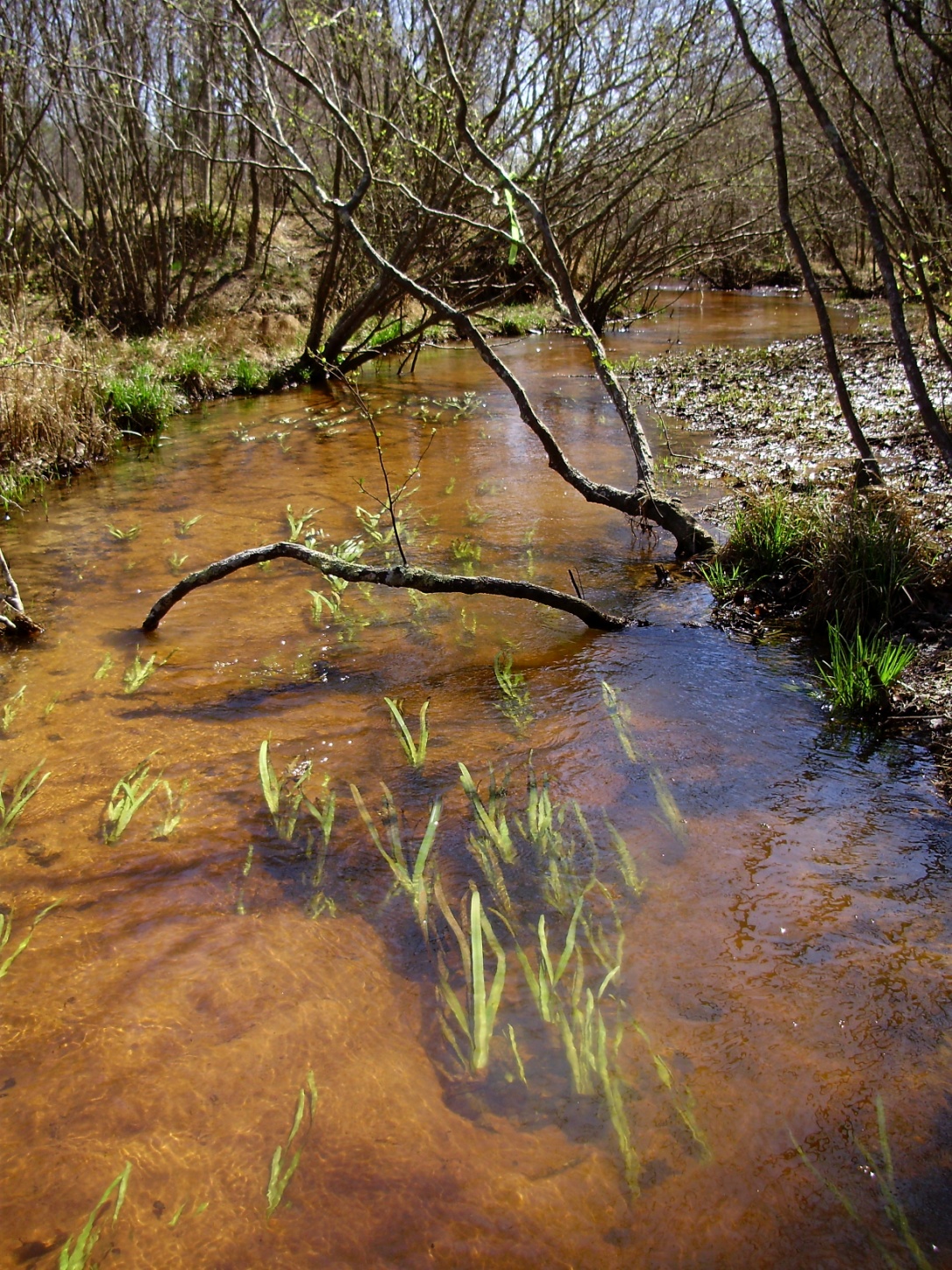


Figure S3. Photograph of a spring run tributary of Puskus Creek showing better quality habitat for Yazoo Darters; such habitat is limited, especially in the Yocona River drainage. Photo credit: Ken Sterling.


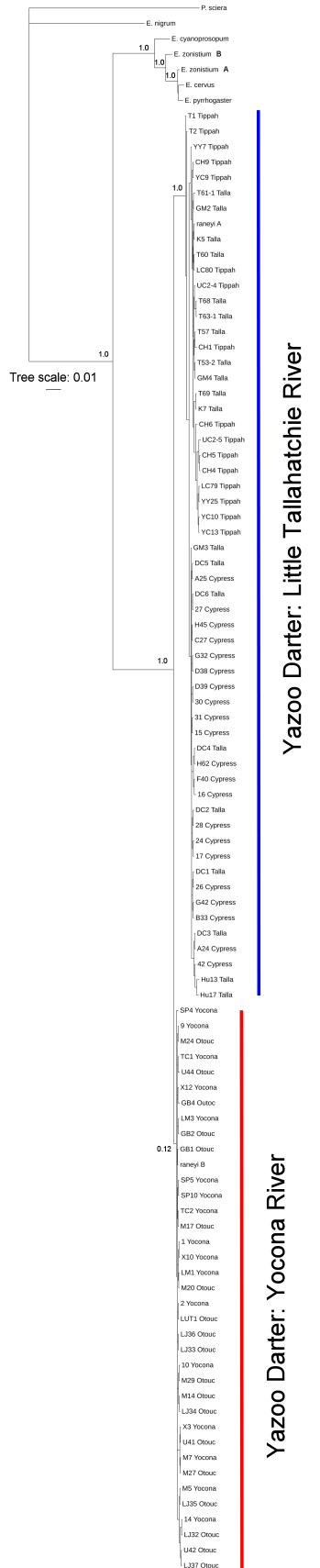


Figure S4. Phylogenetic tree of the partitioned *cytb* dataset using Bayesian estimation (MrBayes ver. 3.2.6) showing Bayesian posterior probabilities at the nodes (see Tables S1 and S2 for sequence data); red bar = Yocona River drainage, blue bar = Little Tallahatchie River drainage.


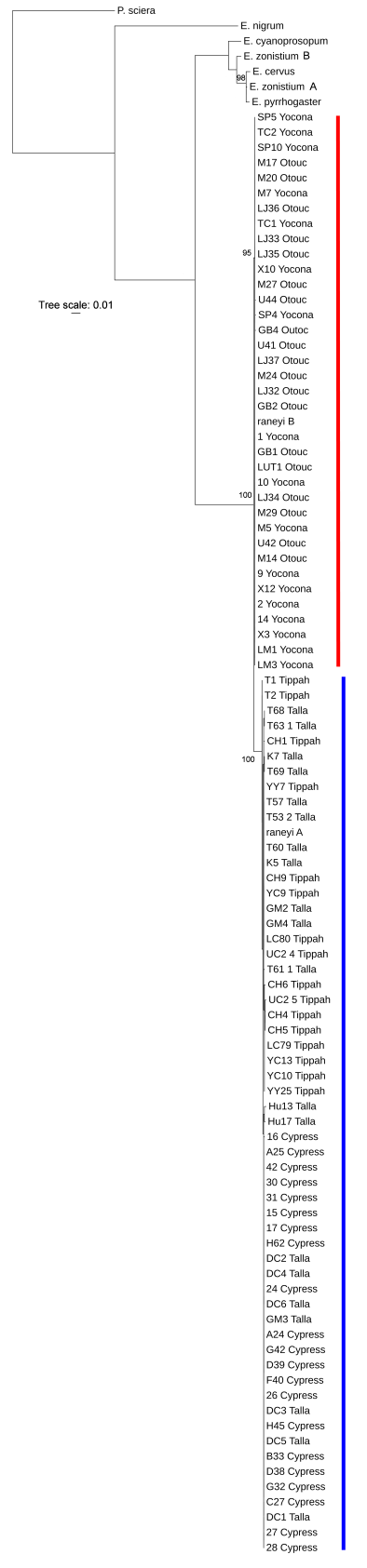


Figure S5. Phylogenetic tree of the partitioned *cytb* dataset using maximum likelihood estimation (RAxML-HPC ver. 8.0) showing bootstrap values at the nodes (see Tables S1 and S2 for sequence data); red bar = Yocona River drainage, blue bar = Little Tallahatchie River drainage.


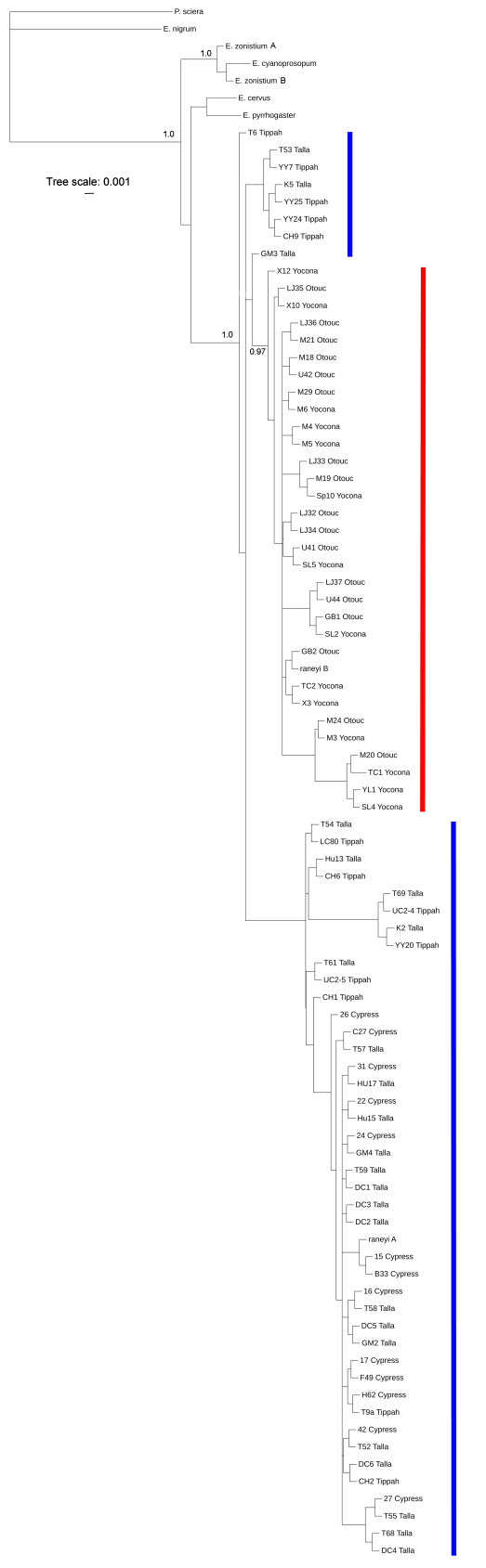


Figure S6. Phylogenetic tree of the partitioned *S7* dataset using Bayesian estimation (MrBayes ver. 3.2.6) showing Bayesian posterior probabilities at the nodes (see Tables S1 and S2 for sequence data); red bar = Yocona River drainage, blue bar = Little Tallahatchie River drainage.


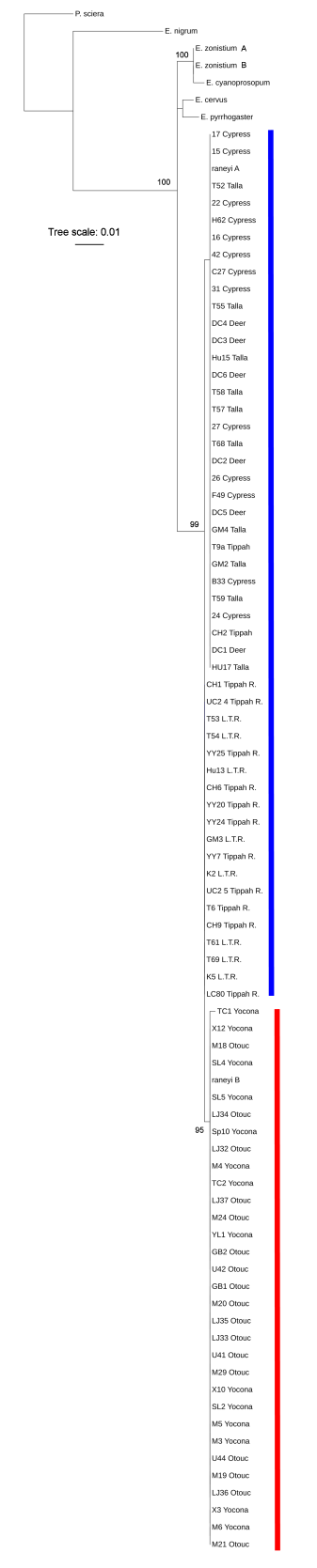


Figure S7. Phylogenetic tree of the partitioned *S7* dataset using maximum likelihood estimation (RAxML-HPC ver. 8.0) showing bootstrap values at the nodes (see Tables S1 and S2 for sequence data); red bar = Yocona River drainage, blue bar = Little Tallahatchie River drainage.

**Literature Cited**

Kozal, L.C., J.W. Simmons, J.M. Mollish, D.J. MacGuigan, E. Benavides, B.P. Keck, and T.J. Near. 2017. Phylogenetic and morphological diversity of the *Etheostoma zonistium* species complex with the description of a new species endemic to the Cumberland Plateau of Alabama. Bulletin of the Peabody Museum of Natural History 58:263-286.

Near, T.J., J.C. Porterfield, and L.M. Page. 2000. Evolution of cytochrome *b* and the molecular systematics of *Ammocrypta* (Percidae: Etheostomatinae). Copeia 2000:701-711.

Near, T.J., C.M. Bossu, G.S. Bradburd, R.L. Carlson, R.C. Harrington, P.R. Hollingsworth, B.P. Keck, and D.A. Etnier. 2011. Phylogeny and temporal diversification of darters (Percidae: Etheostomatinae). Systematic Biology 60:565-595.

Porterfield, J.C. 1998. Phylogenetic systematics of snubnose darters (Percidae: *Etheostoma*), with discussions of reproductive behavior, sexual selection, and the evolution of male breeding color. PhD Thesis, University of Illinois at Urbana-Champaign, Urbana, IL 154 pp.

Powers, S.L., and M.L. Warren Jr. 2009. Phylogeography of three snubnose darters (Percidae: Subgenus *Ulocentra*) endemic to the southeastern US Coastal Plain. Copeia 2009:523-528.

Switzer, J.F. 2004. Molecular systematics and phylogeography of the *Etheostoma variatum* species group (Actinopterygii: Percidae). PhD Dissertation. St. Louis University, St. Louis MO.
